# Supplementary material for: Spatial Variation in Stream-Water Bacterial Communities Across Sites Differing in Wild Chinese Giant Salamander Detection Frequency
Source: Biology (Basel). 2026 Sep 18;15(18):1647. doi: 10.3390/biology15181647 (PMC13604818; doi:10.3390/biology15181647)
Supplement: Supplementary file 1 [file biology-15-01647-s001.zip › Data Related to “Spatial Variation in Stream-Water Bacterial Communities across Sites Differing in Wild Chinese Giant Salamander Detection Frequency”/Microbial Data/result/03.TaxonVisual/heatmap/sample/heatmap.sample.s.cluster.pdf]

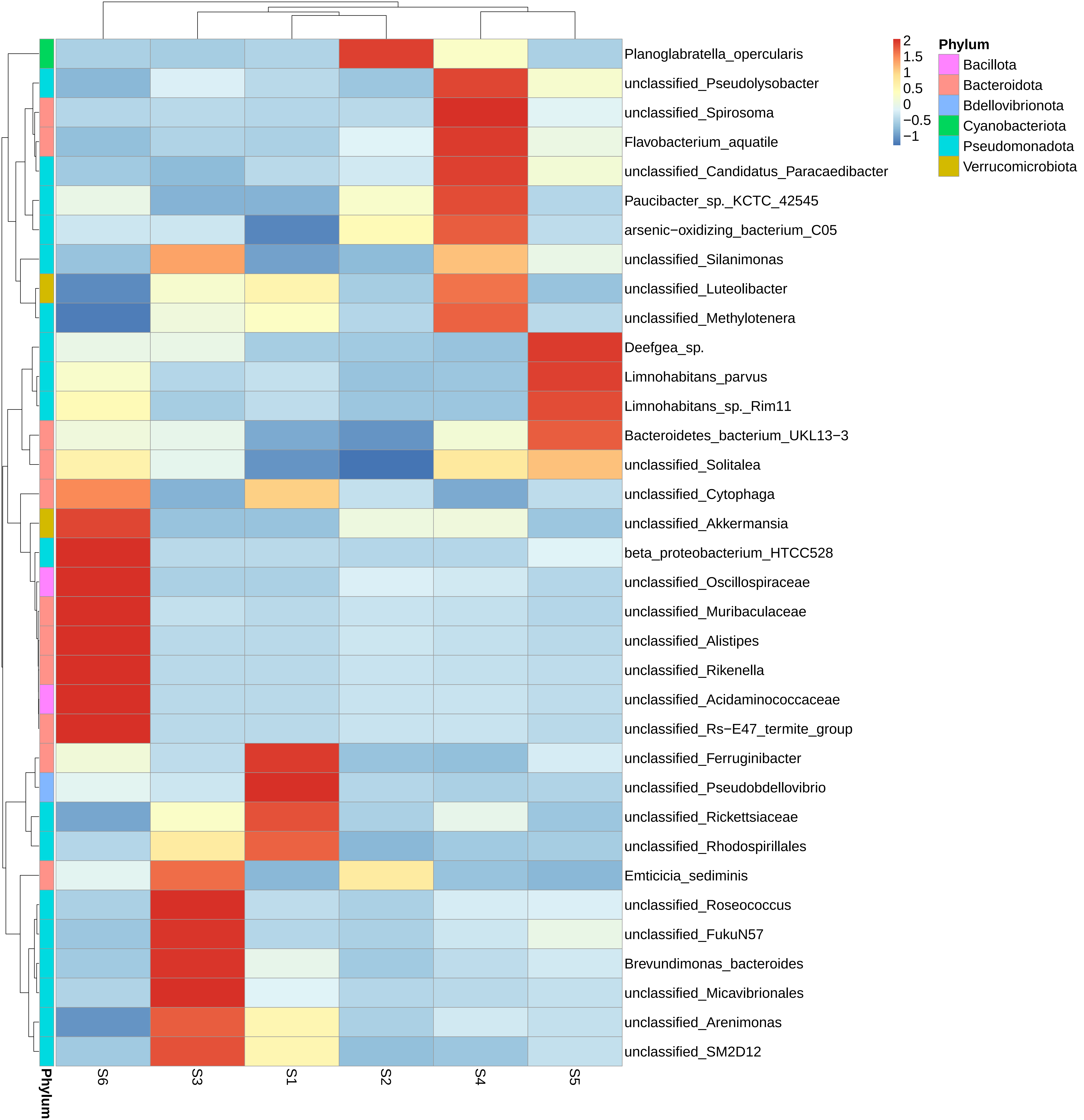

Planoglabratella_opercularis
unclassified_Pseudolysobacter
unclassified_Spirosoma
Flavobacterium_aquatile
unclassified_Candidatus_Paracaedibacter
Paucibacter_sp._KCTC_42545
arsenic−oxidizing_bacterium_C05
unclassified_Silanimonas
unclassified_Luteolibacter
unclassified_Methylotenera
Deefgea_sp.
Limnohabitans_parvus
Limnohabitans_sp._Rim11
Bacteroidetes_bacterium_UKL13−3
unclassified_Solitalea
unclassified_Cytophaga
unclassified_Akkermansia
beta_proteobacterium_HTCC528
unclassified_Oscillospiraceae
unclassified_Muribaculaceae
unclassified_Alistipes
unclassified_Rikenella
unclassified_Acidaminococcaceae
unclassified_Rs−E47_termite_group
unclassified_Ferruginibacter
unclassified_Pseudobdellovibrio
unclassified_Rickettsiaceae
unclassified_Rhodospirillales
Emticicia_sediminis
unclassified_Roseococcus
unclassified_FukuN57
Brevundimonas_bacteroides
unclassified_Micavibrionales
unclassified_Arenimonas
unclassified_SM2D12
S6
S3
S1
S2
S4
S5
Phylum
2
1.5
1
0.5
0
−0.5
−1
Phylum
Bacillota
Bacteroidota
Bdellovibrionota
Cyanobacteriota
Pseudomonadota
Verrucomicrobiota
